# Discrimination and Evaluation of Wild *Paris* Using UHPLC-QTOF-MS and FT-IR Spectroscopy in Combination with Multivariable Analysis

**DOI:** 10.1155/2023/8425016

**Published:** 2023-05-12

**Authors:** Yuangui Yang, Fan Li, Hongbo Xu, Zhishu Tang, Yuanzhong Wang

**Affiliations:** ^1^Shaanxi Collaborative Innovation Center of Chinese Medicine Resources Industrialization, State Key Laboratory of Research & Development of Characteristic Qin Medicine Resources (Cultivation), Shaanxi Innovative Drug Research Center and College of Pharmacy, Shaanxi University of Chinese Medicine, Xianyang 712046, China; ^2^Shaanxi Institute of International Trade & Commerce, Xianyang 712046, China; ^3^China Academy of Chinese Medical Sciences, Beijing 100700, China; ^4^Institute of Medicinal Plants, Yunnan Academy of Agricultural Sciences, Kunming 650200, China

## Abstract

Genus *Paris* has numerous bioactive constituents such as steroid saponins, flavonoids, and polysaccharose which are responsible for antitumor, hemostatic, and anthelmintic, etc. In this study, ultrahigh performance liquid chromatography coupled to time-of-flight mass spectrometer (UHPLC-QTOF-MS) and Fourier transform infrared (FT-IR) spectroscopy in combination with multivariable analysis were employed to discriminate the different species of *Paris* including *P. polyphylla* var. *yunnanensis* (PPY), *P. polyphylla* var. *alba*, *P. mairei* (PM), *P. vietnamensis*, and *P. polyphylla* var. *stenophylla*. Partial least square discriminate analysis based on UHPLC, FT-IR, and midlevel data fusion was used to distinguish 43 batches of Paris. Chemical constituents of different species *Paris* were determined by UHPLC-QTOF-MS. The result indicated that midlevel data fusion had a good performance in the classification compared to a single analytical technology. A total of 47 compounds were identified in different species *Paris*. The similar results indicated that PM could be treated as a proposal substitute of PPY.

## 1. Introduction

Genus *Paris* (Lilaceae family) including 24 species, is mainly distributed in the southwest of China except for *Paris birmanica* and *P. japonica* [1]. As an ethnic medicine, it is recorded in Shennong Materia Medica which is one of the longest medical books in China for treatment of hyperspasmia and bite wound in the folk for a long history [2]. In addition, *P. polyphylla* var. *yunnanensis* (PPY) and *P. polyphylla* var. *chinensis* have been documented in Chinese Pharmacopoeia (ChP) named as Rhizome Paridis (RP) for treating in furunculosis, throat-swelling, traumatic injury, and so on [3]. Modern investigation suggests *Paris* has bioactive constituents such as steroid saponins, flavonoids, and polysaccharose which are responsible for antitumor, hemostatic, and anthelmintic, etc [4, 5]. The extraction of RP is material to make the Chinese patent drugs contain “Gongxuening capsule” and “Jidesheng Sheyao Tablet.” Numerous sources are gradually applied to the industrial production in decade years. Following price of commercial produce including PPY and PPC is higher than the past in the market. Up to now, *P. polyphylla* var. *stenophylla* (PPS), *P. mairei* (PM), and *P. vietnamensis* (PV) are barely investigated by isolation and identification of the major bioactive steroidal saponins [6–8]. At the same time, few research studies could not notice the *P. polyphylla* var. *alba* (PPA). The relationship between different species is hardly illuminated in previous study either.

In previous study, the research indicated an amount of active compounds was found in *Paris*, while only four bioactive markers were determined by ChP for the quality control of RP. It could not respond to multiconstituents and multitargets for traditional Chinese medicine. It was therefore necessary to develop an analytical method which could be responsible for the comprehensive chemical compounds. Fortunately, fingerprints analysis with the advantage of systematic and effective characteristics is used to evaluate the sample of different geographical origin [9, 10], parts [11], and species [12, 13]. As far as we know, chromatographic and spectrographic fingerprints were investigated by previous study. The former could provide the unambiguous and specific information of compounds based on the peak signal. At the same time, the later with the overall and convenient characteristics are used to illustrate the chemical profile. For evaluation of herbal medicine, ultrahigh performance liquid chromatography (UHPLC) as a fast and effective method could provide the targeted compounds of samples [14]. Meanwhile, Fourier transform infrared spectroscopy (FT-IR) with nondestructive and feasible character is a characteristic of integrated chemical information [15]. However, few research studies have been focused on the strategy which combined UHPLC and FT-IR with chemometric for evaluation of Genus *Paris*.

Compared to a single analytical technique, data fusion of different instrumental (spectroscopic and chromatographic) techniques together with multivariate chemometrics can increasethe model classification [16]. Three date fusion strategies (low-, mid-, and high level) can be carried out basically. Especially, mid-level fusion can be able to filter block noise and enables interpretation of the results, firstly extracts some relevant features from each data of instrument, and then merges them into a single array that is used for classification [17]. The aim of this investigation is to validate and develop an analytical strategy which can evaluate and discriminate different species of *Paris* with regard to PPY, PPA, PPS, PM, and PA. Firstly, chemical profiling of *Paris* was identified by UHPLC-QTOF-MS. Then, different species of *Paris* were discriminated through UHPLC, FT-IR, and data fusion coupled to chemometrics. It is expect that the study can provide a fundamental of genus *Paris* sources.

## 2. Experiment

### 2.1. Materials and Reagents

**Figure 1:**
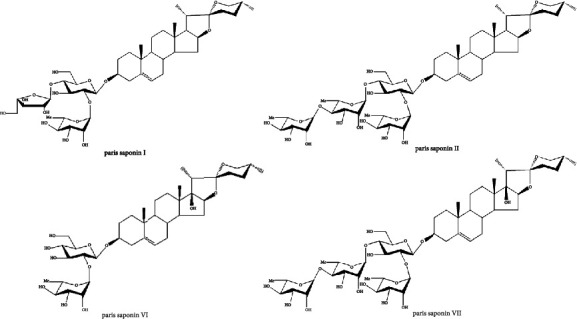
Chemical structures of four bioactive markers PSI, PSII, PSVI and PSVII.

Forty-three samples of the fresh five species of Genus *Paris* were collected from different regions in Yunnan Province Southwestern China and were identified by Professor Jinyu Zhang in Yunnan Academy of Agricultural Sciences. The detailed information of samples was listed in Table 1. Voucher specimens were deposited in Institute of Medicinal Plants, Yunnan Academy of Agricultural Sciences. Standard compounds *Paris* saponin I (PSI, No: MUST-21110718) and *Paris* saponin II (PSII, No: MUST-21062804) were purchased from Chengdu Must Biotechnology Co., Ltd. (Chengdu, China). *Paris* saponin VI (PSVI, No: PRF20080742) and *Paris* saponin VII (PSVII, No: PRF20121241) were provided by Chengdu Biopurify Biotechnology Co., Ltd. The purity of all reference compounds was determined to be over 98% by UHPLC. The structures of standard constituents were displayed in Figure 1.

HPLC-grade acetonitrile and formic acid were provided by Dikmapure Co., Ltd. (Lake Forest, MA, USA), respectively. Distilled water was further purified by a Milli-Q system (Bedford, MA, USA). Analytical grade methanol was purchased from Kemiou Chemical Reagent Co., Ltd. (Tianjin, China). Supersonic wave cleaning equipment (SY3200-T) was purchased from Hengda Ultrasonic Equipment Co., Ltd. (Zhejiang, China). Spectroscopic grade potassium bromide (KBr) was purchased from Fengchuan Fine Chemical Research Institute (Tianjin, China).

### 2.2. Apparatus

A UHPLC-UV system (Shimadzu, Kyoto, Japan) equipped with a degasser, binary gradient pumps, UV detector, a column oven, and an auto-sampler was utilized to obtain UHPLC fingerprints. System control and data analysis were conducted by LabSolution software (Shimadzu). The chromatographic separation was operated on Shim-pack XR-ODS III column (150 × 2.0 mm, 2.2 *μ*m) with UV detector where the detection wavelength was set at 203 nm. The mobile phase was (A) 0.05% formic acid in water and (B) acetonitrile with a gradient program as follows: 17% B, 0–1.5 min; 17–23% B, 1.5–4.0 min; 23% B, 4.0–8.7 min; 23–38% B, 8.7–18 min; 38-60% B, 18-25.6 min; 60–17% B, 25.6–28 min; 28–32 min, 17%. In order to identify the chemical constituents of *Paris*, gradient program for UHPLC-QTOF-MS was 0–2 min, 17% B; 2–5 min, 17%–23% B; 5–10 min, 23%–40% B; 10–35.5 min, 40%–60% B; 35.5–38 min, 60%–95% B; 38–40 min, 95%; 40-41 min, 19% B. Other chromatographic parameters were as follows: The column temperature was maintained at 45°C. The flow rate was 0.45 mL/min. All samples were injected with 1 *μ*L.

Mass spectrometry was performed on a Triple-TOFTM 5600^+^ system mass spectrometer (AB SCIEX, Foster City, CA, USA). Data acquisition was conducted in the negative electrospray (ESI) ionization mode. The ESI-MS parameters were as follows: mass scan range for both TOF-MS and TOF-MS/MS: m/z 50–1200, ion spray voltage: 4500 V, atomizer temperature (TEM): 500°C; declustering potential (DP): 80 V; curtain air pressure (CUR): 40 psi; nebulize gas (Gas 1) and auxiliary gas (Gas 2) pressure: 50 psi. The collision energy was set at 50 eV. Instrument control, data acquisition, and analysis of data were Analyst TF 1.6 and PeakView 1.2.

For FT-IR, table press (Shanghai, China) was employed to press the samples into thin sample. FT-IR (Perkin-Elmer, Norwalk, CT, USA) was equipped with a deuterated triglycine sulfate detector. IR spectra were recorded from the accumulation of 16 scans in 4000–400 cm^−1^ range with a resolution of 5 cm^−1^.

### 2.3. Sample Preparation

For UHPLC, 0.1 g of sample powder after passing through a 60 mesh sieve was weighed accurately, transferring into 10 mL glass stopper tube and adding 2 mL of 80% methanol with ultrasonically extracted for 40 min at room temperature. The filtrates were collected by filter paper after sample solution cooling to the room temperature. All solution was stored at 4°C and passed through a 0.22 *μ*m membrane filter before injection into the UHPLC system. The standard stock solution for qualitative analysis was dissolved all reference standards with methanol to final concentration of 1 mg/mL. For FT-IR, 1.5 mg of dried sample and 100 mg KBr was weighted accurately and mixed completely by the agate mortar and then a homogeneous tablet was pressed . The KBr pellet background of CO_2_ and H_2_O was deducted before experiment. The FT-IR spectra of sample were scanned 2 times and recorded after preheating 60 min under relative humidity 65% at the temperature 25°C.

### 2.4. Data Analysis

UHPLC chromatographic fingerprints were recorded by the sampling points from 2.5 to 29 min with the time interval of 0.0833 min. Owing to the disturbance of methanol signal noise, the sampling points were rejected before 2.5 min. Date of UHPLC was entered into *Similarity Evaluation System for Chromatographic Fingerprint of Traditional Chinese Medicine* (Version 2004A, Chinese Pharmacopoeia Committee) for similarity analysis. For FT-IR, the data of spectra were entered into the software of OMNIC (Version 8.2, Thermo Fisher Scientific Inc, USA), which were used by noise reduction, baseline correction, and so on.

As a supervised method, PLS-DA is used to concentrate the multidimension data into two dimension, which can illustrate a sample whether it belong to a special class [18]. To evaluate and discriminate *Paris* of different species, PLS-DA based on UHPLC and FT-IR spectra was used to make the further study. All of data were imported into SIMCA-P^+^ 10.0 (Umetrics AB, Umea, Sweden). To remove the baseline shifts and overlap peaks, the data were subjected to second derivative before analysis [19].

## 3. Results and Discussion

### 3.1. Method Validation

For UHPLC, steroid saponins including PSI, PSII, PSVI, and PSVII were used to develop and validate method for UHPLC. Precision was performed by the inter- and intraday variations to make the analytical method accurate. The intraday was accumulated by retention time and peak areas using six repetitive injection of mixed standards in a day and three consecutive days for the interday variation. The results implied that relative standard deviations (RSD) of intra- and interday were no more than 2.98% and 2.83%, respectively (Table 2).

For FT-IR, the precision was performed using five consecutive scan with a sample, and the results indicated that the correlation coefficient was ranged 0.9996–0.9999 with RSD = 0.05%. Stability Stability was calculated by scanning a sample every ten minutes and five consecutive times. The results suggested that correlation coefficient was between 0.9995 and 0.9999, and RSD = 0.03%. Repeatability was carried out by five tablets with a sample for scanning, and the results implied that correlation coefficient was more than 0.9996, and RSD = 0.02%.

### 3.2. Similarity Analysis for UHPLC

**Figure 2:**
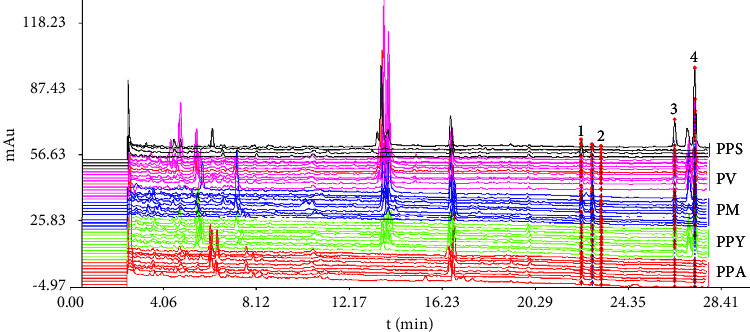
UHPLC fingerprints of 43 batches different species of *Paris*, peaks “1, 2, 3 and 4” represent PSVII, PSVI, PSII and PSI.

**Figure 3:**
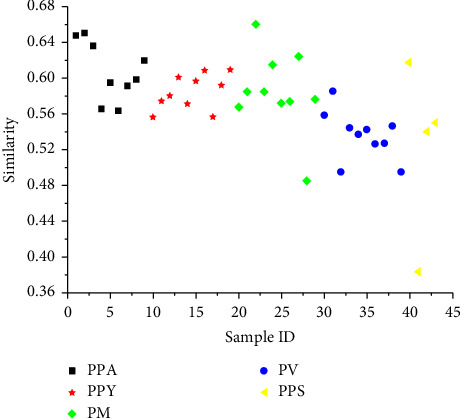
Similarity values based on UHPLC for *Paris*.

UHPLC chromatographic fingerprint of 43 batches five species of *Paris* is shown in Figure 2. Peaks 1, 2, 3, and 4 in chromatographic pattern were assigned the bioactive compounds of PSVII, PSVI, PSII, and PSI based on the reference standards. As we can see, PSII and PSI changed significantly in each sample with even not being detected in PPA, whereas PSVI was found in the most of samples. It indicated that chemical constituents varied markedly for the *Paris* of different species. The result was similar to the previous research that the contents of chemical components have difference among collections of *Paris* of different geographical origins and species [20]. For similarity evaluation, the average chromatogram of 43 batches of samples was treated as the standard characteristic fingerprint. Similarity values were calculated by compared with each chromatogram of the *Paris* samples to the average chromatogram. As shown in Figure 3, the similarities by all of samples were no more than 0.66 (PM3). For the most of samples, the values were ranged from 0.55 to 0.65. The changed chemical constituents of sample may be discovered in PM9 (0.49) and PPS2 (0.38) with the value less than 0.5. However, it is difficult to clarify the relationship between different species of *Paris*.

### 3.3. Discrimination of *Paris* by UHPLC

**Figure 4:**
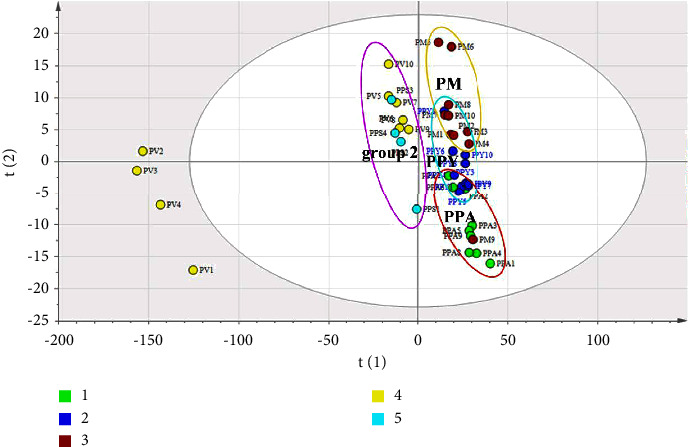
Two-dimension score plot of PLS-DA based on UHPLC for PPA, PPY, PM, PV and PPS.

As shown in Figure 4, a two-dimension score scatter plot with 95% confidence ellipses of PLS-DA was used to evaluate and discriminate the different species of *Paris*. It is significant among various sample PV from Simao City of Yunnan Province with four outliers PV1, PV2, PV3, and PV4. The collection sample with multiple soil, moisture, and surrounding environment may lead to vary in chemical components of each sample [9]. The phenomenon is necessary to work in the further study. It is obvious that the chemistry in each sample changed markedly for the similar species in the rhizome of PV. Moreover, group 1 including PPA, PPY, and PM was distributed in the positive principle compound (PC) 1; on the contrary, other species of *Paris* in the negative PC1 were assigned group 2. The previous morphological research reported that PPA, PPY, and PPS belong to the varieties of *P. polyphylla* Smith [1]. The similar profile was found according to the theory of macroscopical phenotype. Fortunately, it is associated with the result that is a close relationship between PPA and PPY compared to other species, especially PPA2, PPA6, PPA7, and PPA10 which are close to all of PPY species except for PPY2. However, the species of PPS had tight distance to PV without macroscopical theory. In group 1, *P. marirei* was closely related to *P. polyphylla* Smith, particularly in the species of PPY. As mentioned above, it is indicated that the chemical profile was usually related to the macroscopical characteristic with the different *Paris*. The similar report in the previous research shows that chemotaxonomic studies of nine *gentianaceae* indicate some secondary metabolites could be treated as potential chemotaxonomic markers to differentiate *gentianaceae* species. Moreover, the plots were weakly loose among the same species in the scatter plot which indicated that the chemical profile varied notably among the most of samples. As an official medical resource, PPY has been widely applied to industrial production and clinic. Due to long growth cycle and excessive excavation, the wild population of PPY is gradually decreased. Obviously, since they had the similar chemical profile, PPA and PM could be treated as a proposal substitute of PPY. UHPLC was used to evaluate and discriminate different species of *Paris* with the characteristic of rapid and accurate compared with other technology. However, the chromatographic pattern may not be responsible for the entire bioactive chemistry of sample. A convenient and nondestructive method by FT-IR is necessary for distinguishing *Paris*.

### 3.4. Discrimination of *Paris* by FT-IR

**Figure 5:**
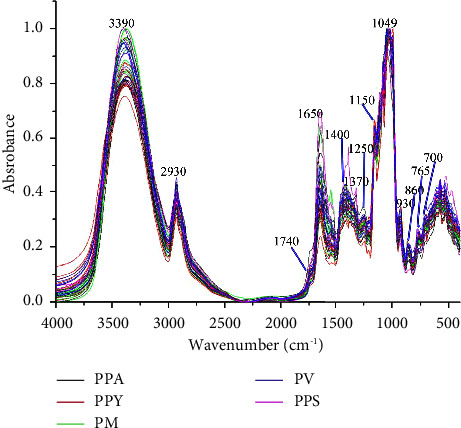
FT-IR spectroscopy of 43 batches different species of *Paris*.

FT-IR is a technique based on the absorbance of light from 4000 to 400 cm^−1^, which has been widely used for evaluation of herbal medicine and authentication of food products [21]. In this study, the samples of different species of *Paris* were analyzed by the FT-IR spectra shown in Figure 5. The similar position and shape of peak were found in the most of sample. The peaks were assigned as following: 3390, 2930, 1740, 1650, 1400, 1370, 1250, 1150, 1049, 930, 860, 765, and 700 cm^−1^, which are characteristic common peaks of spectra. The wavelength at 3390 cm^−1^, generally the most prominent peak, was due to the stretching vibration peak of O-H in sugar moiety. The peak at 2930 cm^−1^ was assigned to the stretch of methylene group. Absorption band at around 1740 and 1650 cm^−1^ corresponding to bending C=O and C=C stretching, respectively, was potentially related to steroid saponins, flavonoids, and fatty acids. The peaks at 1400 and 1370 cm^−1^ were due to plane deviational vibrations of methylene or methyl group. A bond of 1250 cm^−1^ was responded to the vibration peak of C-O in alcohol hydroxyl group. In addition, according to the previous research that peaks at 930, 860, 1150, and 1049 cm^−1^, these were due to steroidal saponins skeletal vibration [22]. As mentioned above, the similar reported study shows that the main constituents were steroids saponins in *Pairs*, others including flavonoids, fatty acids, and so on [5].

**Figure 6:**
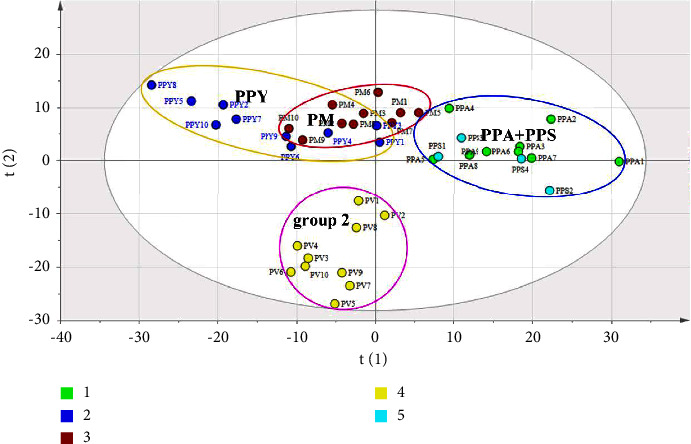
Two-dimension score plot of PLS-DA based on FT-IR for PPA, PPY, PM, PV and PPS.

As shown in Figure 6, the two-dimension score plots were carried out by FT-IR in conjunction with PLS-DA for evaluation of different species of *Paris*. The result agreed with the analysis of UHPLC that PPY, PPA, and PM belonged to group 1 in positive PC2, and PV was attributed group 2 in negative PC2. In addition, the same phenomenon found that PPY was more closely related with PM than with PPA. However, the various results show that PPS was located in group 1 for this investigation. As the varieties of *P. polyphylla* Smith, PPS had the similar chemical constituents while the various contents were compared with UHPLC. It could draw a conclusion that the varieties of *P. polyphylla* including PPY and PPA were closely related with *P. marirei* no matter what analytical technology UHPLC or FT-IR held.

### 3.5. Discrimination of *Paris* by Midlevel Data Fusion

**Figure 7:**
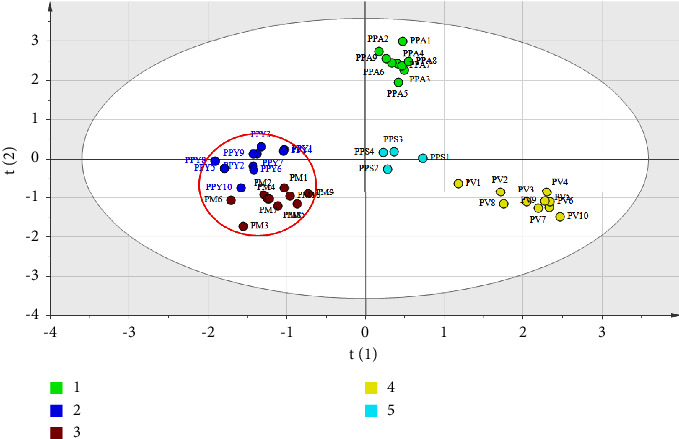
Two-dimension score plot of PLS-DA based on mid-level data fusion for PPA, PPY, PM, PV and PPS.

Midlevel data fusion for PLS-DA was used to discriminate *Paris* of different species. The combination of relevant features based on UHPLC and FT-IR was applied to establish a model which could be used for synthetic evaluation of *Paris*. As show in Figure 7, two dimension score PLS-DA of midlevel data fusion is displayed. Five species of *Paris* had a good performance for classification in the data fusion compared to a single instrumental data. Interestingly, it was similar to a single data model that PPY and PM had a close relation. It demonstrated that PM as a substitute of PPY with the further study was used to solve the resource shortage. In addition, all of PPA and PPS were located in the positive PC1 and PC2 except for PPS2. The chemical profile was related to macroscopical characteristic which was proved in this study.

### 3.6. Chemical Analysis of *Paris* Using UHPLC-QTOF-MS

The mix sample of different species *Paris* was determined by UHPLC-Q-TOF MS in the negative electrospray modes. The references PSI, PSII, PSVI, and PSVII were classified, and the cleavage patterns were summarized. In addition, the characteristic ionic fragments of compounds were summarized based on cleavage fragments and structures of *Paris*. Finally, the compounds were identified by molecular weight and secondary fragmentation. A total of 47 compounds were identified in different species *Paris*. As shown in Table 3, it included twenty-three isosproterenol saponins, five furostanol saponins, four cholestanol saponins, four flavonoids, three pentacyclic triterpenoids, two C21 steroids, two phytosterol saponins, and four others.

As the main type of Paris saponins, isoproterenol saponins included pennogenin and diosgenin type. Sixteen pennogenin saponin including peaks 1, 5, 6, 7, 10, 11, 13, 16, 17, 19, 21, 28, 29, 43, 44, and 45 were identified. We take peak 16 and 39 as an example for fragmentation pathway of pennogenin saponins. The parent ion at m/z 1107.4873 [M + COOH]-of peak 6 lost a xylose to generate m/z 929.4470, and the characteristic fragment ion of m/z 439.2541 was further generated through losing a rhamnose, a xylose, and a glucose. Peak 16 was unambiguously identified as parisyunnanoside H. Peak 39 exhibited deprotonated ion at m/z 1029.5424 losing three rhamnose and a glucose to generate m/z 429.1363. Peak 18 was identified as polyphyllin VII according to structure, molecular formula, fragment ions, and reference. Seven diosgenin saponins including peaks 20, 23, 24, 25, 35, 39, and 42 were identified. Peak 42 showed precursor ion [M-H]- at m/z 1013.5392 and product ion at m/z 867.4844 [M-H-Rha], m/z 721.4232 [M-H-2Rha], m/z 575.3578 [M-H-3Rha], and m/z 413.2257 [M-H-3Rha-Glc]. Peak 42 was identified as polyphyllin II according to structure, fragment ions, and reference. Peak 44 provides [M-H]-at m/z 853.4645 and lose a rhamnose, an arabinose, and a glucose to generate diagnostic ion of diosgenin saponin. It was identified as polyphyllin I.

Furostenol saponins was an F-ring opening, which always connected with glucose at C_3_-OH and C_26_-OH. We take peak 26 as an example for fragmentation pathway of furostenol saponins. It provides [M-H]-at m/z 1063.50417 and lose a rhamnose and a galactose to generate fragment ions m/z 917.4845 and m/z 755.4325, then lose two glucose at C-3 and C-26 to generate diagnostic ion at m/z 431.3246. Peak 26 was identified as polyphyllin H. In addition, cholestanol saponins, flavonoids, and other types were discovered in different species of *Paris*. In negative mode, myricetin (peak 41) was identified due to parent ion m/z 313.2410 losing a carbonyl group to generate m/z 285.2263. Peak 4 provides deprotonated ion at m/z 479.3019 which was dehydrated to generate fragment ion at m/z 461.2961 and lose C_7_H_14_O_4_ to produce m/z 319.1921, then dehydrated finally to generate m/z 283.1725. It was identified as *β*-ecdysone.

**Figure 8:**
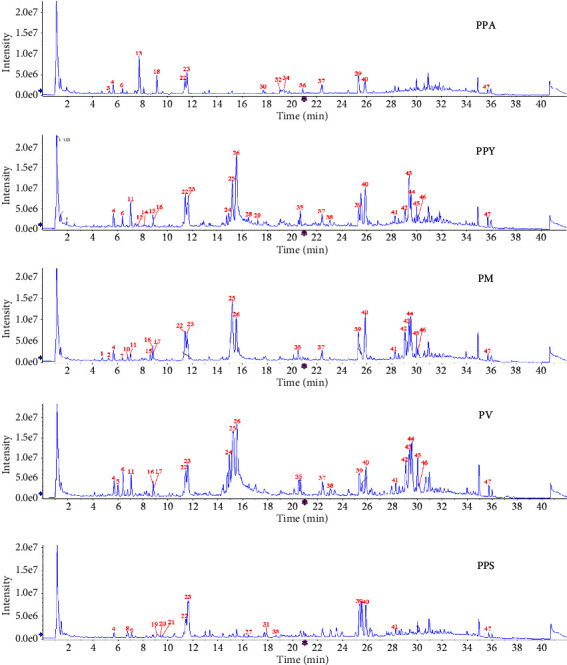
Chromatography of PPA, PPY, PM, PV and PPS using UHPLC-QTOF-MS.

In comparison of five species *Paris*, PPY, PM, and PV had the similar chemical constituents, especially for PM (Figure 8). The result agreed with the previous study UHPLC, FT-IR spectroscopy and mid-data fusion in combination with multivariable analysis. The common peaks of PPY and PM are peak 4 (*β*-ecdysone), peak 11 (Parisyunnanoside G isomer), peak 16 and 17 (Parisyunnanoside H isomer), peak 22 (Polyphyllin G), peak 25 (Pennogenin-3-O-Glc-(1-5)-Ara(1-4)[Rha(1-2)]-Glc), peak 26 (Polyphyllin H), peak 35 (Parisaponin I), peak 37 (glyceryl *α*-mono-palmitate), peak 39 (Polyphyllin VII), peak 40 (Cussonoside B), peak 41 (*Paris* saponins VI), peak 42 (Polyphyllin II), peak 43 (Polyphyllin III), peak 44 (Polyphyllin I), peak 45 (Polyphyllin V), peak 46 (7-O-Rha-kaempferol-3-O-Glc), and peak 47 (4,2′,4′-trihydroxy-chalcone).

## 4. Conclusions

To the best of our knowledge, the wild *Paris* was gradually decreasing due to the industrial production. The aim of this study was to distinguish Genus *Paris* including PPA, PPY, PM, PV, and PPS by using UHPLC, FT-IR and midlevel data fusion in combination with multivariate analysis. Data fusion could classify the sample better than a single data array. Chemical constituents of different species *Paris* were determined by UHPLC-Q-TOF MS. The similar results showed that chemical constituents of PM were similar to PYY. It is expected that PM as a substitute of PPY with the further study was used to solve the resource shortage.

## Figures and Tables

**Table 1:** Information of 43 bacthes six species of *Paris*.

Code	Number	Species	Site
PPA1-PPA9	9	*P. polyphylla* var. *alba*	Hani-Yi autonomous prefecture of Honghe of Yunnan province
PPY1-PPY10	10	*P. polyphylla* var. *yunnanensis*	Baoshan city of Yunnan province
PM1-PM10	10	*P. mairei*	Lijiang city of Yunnan province
PV1-PV10	10	*P. vietnamensis*	Pu'er city of Yunnan province
PPS1-PPS4	4	*P. polyphylla* var. *stenophylla*	Zhaotong city of Yunnan province

**Table 2:** Intra- and inter-day of four standards.

	Intra-day (*n* = 6)		Inter-day (*n* = 3)	
	*t* (%)	*P* (%)	*t* (%)	*P* (%)
PSI	0.06	1.36	0.11	2.57
PSII	0.06	2.98	0.12	2.83
PSVI	0.02	2.28	0.11	2.64
PSVII	0.03	1.94	1.21	2.06

“*t*” the retention time of common peak of standard; “*P*” the peak areas of common peak of standard.

**Table 3:** Chemical constituents of PPA, PPY, PM, PV and PPS using UHPLC-QTOF-MS.

Peak	Compound name	Type	Observed m/z	*t*_(*Rt*)_	Mass fragment
1	Parisyunnanoside G isomer	Isospirosteranol	1223.585 [M-H]^−^	4.775	1077.5146 [M-H-Rha]^−^, 931.4593 [M-H-2Rha]^−^, 769.4178 [M-H-2Rha-Gal]^−^, 641.3581 [M-H-2Rha-Gal-C_5_H_4_O_4_]^−^, 479.3058 [M-H-2Rha-Gal-Glc-C_5_H_4_O_4_]^−^
2	Paritriside A	Pentacyclic triterpenoids	765.1597 [M-H]^−^	5.277	737.1350 [M-H-CO]^−^, 633.1602 [M-H-CO-Ara]^−^, 455.1043 [M-H-COO-Ara-Glc]^−^
3	Pseudoproto-Pb	Furostanol	1079.4931 [M-H]^−^	5.345	948.4591 [M-H-Xyl]^−^, 623.1659 [M-H-Xyl-2Glc]^−^
4	*β*-ecdysone	Cholestanol	479.3019 [M-H]^−^	5.696	461.2961 [M-H_2_O]^−^, 319.1921 [M-C_7_H_14_O_4_]^−^, 301.1824[M-C_7_H_14_O_4_-H_2_O]^−^, 283.1725 [M-C_7_H_14_O_4_-2H_2_O]^−^
5	24-O-Gal-(23*S*,24*S*)-spirosta-5,25 (27)-diene-1*β*,3*β*,23,24-tetrol-1-O-Xyl (1-6)-Glc (1-3)[Rha (1-2)]-Glc isomer	Isospirosteranol	1223.5473 [M-H]^−^	6.024	1091.5052 [M-H-Xyl]^−^, 945.4456 [M-H-Xyl-Rha]^−^
6	24-*O*-Gal-(23*S*,24*S*)-spirosta-5,25 (27)-diene-1*β*,3*β*,23,24-tetrol-1-O-Xyl (1-6)-Glc (1-3)[Rha (1-2)]-Glc	Isospirosteranol	1223.548 [M-H]^−^	6.397	1091.5152 [M-H-Xyl]^−^, 945.4583 [M-H-Xyl-Rha]^−^
7	Parisyunnanoside G	Isospirosteranol	1269.5503 [M + COOH]^−^	6.418	1091.5026 [M-H-Xyl]^−^, 945.4307 [M-H-Xyl-Rha]^−^, 799.3541 [M-H-Xyl-Rha-Fuc]^−^, 637.3417 [M-H-Xyl-Rha-Fuc-Glc]^−^
8	Kaempferol-3-O-Glc (1-4)-Glc	Flavonoids	610.2859 [M-H]^−^	6.799	448.2910 [M-H-Glc]^−^
9	7*β*-ol-sitosterol-3-O-Glc	Phytosterol	592.2584 [M-H]^−^	7.117	564.2737, 548.2576, 515.1946, 119.0345,
10	Padelaoside B	Isospirosteranol	1355.59 [M-H]^−^	6.857	1224.6083 [M-H-Xyl]^−^, 1078.5370 [M-H-Xyl-Rha]^−^, 897.4143 [M-H-Xyl-Rha-OGlc]^−^
11	Parisyunnanoside G isomer	Isospirosteranol	1223.545 [M-H]^−^	7.057	1091.4920 [M-H-Xyl]^−^, 945.5193 [M-H-Xyl-Rha]^−^
12	Parisyunnanoside J	C21 steroids	977.4307 [M-H]^−^	7.619	845.3843 [M-H-Rha]^−^, 797.3667 [M-H-Rha-C_2_H_8_O]^−^, 665.3212 [M-H-2Xyl]^−^, 519.2603 [M-H-2Xyl-Rha]^−^
13	Parisyunnanoside H	Isospirosteranol	1061.4873 [M-H]^−^	7.759	929.4440 [M-H-Xyl]^−^, 783.3871 [M-H-Xyl-Rha]^−^, 637.3271 [M-H-Xyl-Rha-Fuc]^−^
14	Unkonwn 1	Others	479.5069 [M-H]^−^	8.138	461.2921 [M-H_2_O]^−^, 319.1955 [M-C_7_H_14_O_4_]^−^, 301.1837 [M-C_7_H_14_O_4_-H_2_O]^−^, 283.1706 [M-C_7_H_14_O_4_-2H_2_O]^−^
15	Smilaxchinoside B	Furostanol	1195.535 [M-H]^−^	8.64	1049.4931 [M-H-Rha]^−^, 903.4696 [M-H-2Rha]^−^, 741.4197 [M-H-2Rha-Glc]^−^, 579.2568 [M-H-2Rha-2Glc]^−^
16	Parisyunnanoside H isomer	Isospirosteranol	1061.4898 [M-H]^−^	8.816	929.4470 [M-H-Xyl]^−^, 765.3738 [M-H-Xyl-Gal]^−^, 619.3119 [M-H-Xyl-Rha-Gal]^−^, 601.3033 [M-H-Xyl-Gal-Rha-OH]^−^, 439.2532 [M-H-Xyl-Rha-Gal-Glc-OH]^−^
17	Parisyunnanoside H isomer	Isospirosteranol	1107.4977 [M-H]^−^	8.846	929.4452 [M-H-Xyl]^−^, 765.3761 [M-H-Xyl-Gal]^−^, 619.3161 [M-H-Xyl-Rha-Gal]^−^, 439.2543[M-H-Xyl-Rha-Gal-Glc-OH]^−^
18	Polyphyllin H isomer	Furostanol	1109.5073 [M + COOH]^−^	9.17	931.4595 [M-H-Xyl]^−^, 785.4023 [M-H-Xyl-Rha]^−^,623.3481 [M-H-Xyl-Rha-Glc]^−^, 477.2890 [M-H-Xyl-2Rha-Glc]^−^
19	Parisyunnanoside G isomer	Isospirosteranol	1223.5794 [M-H]^−^	9.224	1139.5201 [M-H-C_5_H_14_]^−^, 977.4672 [M-H-C_5_H_14_-Glc]^−^
20	Polyphylloside IV	Isospirosteranol	1061.5255 [M-H]^−^	9.451	916.4755 [M-H-Rha]^−^, 770.4132 [M-H-Rha]^−^
21	Chonglouoside SL-18	Isospirosteranol	931.4614 [M-H]^−^	9.636	903.4320 [M-H-CO]^−^, 757.3472 [M-H-CO-Rha]^−^
22	Polyphyllin G	Furostanol	1049.524 [M-H]^−^	11.445	903.4645 [M-H-Rha]^−^, 757.3544 [M-H-2Rha]^−^, 595.3110 [M-H-2Rha-Glc]^−^, 433.1617 [M-H-2Rha-2Glc]^−^
23	Polyphylloside IV isomer	Isospirosteranol	1061.5263 [M-H]^−^	14.781	929.4769 [M-H-Gal]^−^, 765.4073 [M-H-Gal-Rha]^−^, 619.0835 [M-H-Gal-2Rha]^−^, 439.1617 [M-H-Gal-2Rha-OGlc]^−^
24	Polyphylloside III/parisaponin I	Isospirosteranol	1093.5511 [M + COOH]^−^	14.953	901.4830 [M-H-Rha]^−^, 755.4256 [M-H-2Rha]^−^, 593.3341 [M-H-2Rha-Glc]^−^
25	Pennogenin-3-O-Glc-(1-5)-Ara (1-4) [Rha (1-2)]-Glc	Isospirosteranol	1033.5297 [M-H]^−^	15.231	901.4856 [M-H-Ara]^−^, 755.4224 [M-H-Ara-Rha]^−^, 593.3732 [M-H-Ara-Rha-Glc]^−^, 431.1308 [M-H-Ara-Rha-2Glc]^−^
26	Polyphyllin H	Furostanol	1063.5417 [M-H]^−^	15.551	917.4765 [M-H-Rha]^−^, 901.4845 [M-H-O-Rha]^−^, 755.4325 [M-H-O-2Rha]^−^, 593.3737 [M-H-O-2Rha-Glc]^−^, 431.3246 [M-H-O-2Rha-2Glc]^−^
27	Parispolyside E	Cholestanol	1073.5248 [M + COOH]^−^	16.493	893.4583 [M-H-Ara]^−^, 747.3036 [M-H-Ara-Rha]^−^, 585.3418 [M-H-Ara-Rha-Glc]^−^
28	Chonglouoside SL-3	Isospirosteranol	1063.539 [M-H]^−^	16.524	901.4854 [M-H-Glc]^−^, 755.4158 [M-Glc-Rha]^−^, 609.3327 [M-Glc-2Rha]^−^, 447.2354 [M-2Glc-2Rha]^−^
29	Chonglouoside SL-4	Isospirosteranol	1063.5428 [M-H]^−^	17.274	901.4424 [M-H-Glc]^−^, 755.3874 [M-Glc-Rha]^−^, 609.3722 [M-Glc-2Rha]^−^
30	Parispolyside E isomer	Cholestanol	1027.419 [M-H]^−^	17.72	929.4458 [M-H-C_5_H_12_O]^−^, 783.3883 [M-H-C_5_H_12_O-Rha]^−^, 637.3274 [M-H-C_5_H_12_O-2Rha]^−^,473.2534 [M-H-C_5_H_12_O-2Rha-Glc]^−^
31	3*β*-ol-oleane-12-en-28-oic acid- 3-O-Glc (1-2)-Glc	Pentacyclic triterpenoids	845.3762 [M + COOH]^−^	17.95	637.3279 [M-H-Glc]^−^, 475.3160 [M-H-2Glc]^−^
32	Parispseudoside B	Cholestanol	893.2173 [M-H]^−^	19.085	747.2188 [M-H-Rha]^−^, 585.1578 [M-H-Rha-Glc]^−^
33	Hypoglaucin H	C21 steroids	767.2189 [M-H]^−^	19.101	621.4030 [M-H-Rha]^−^
34	Glyceryl *α*-mono-palmitate	Others	329.2345 [M-H]^−^	19.303	247.2143, 229.1468, 211.1371, 171.1031, 139.1144
35	Parisaponin I	Isospirosteranol	1047.5331 [M-H]^−^	20.677	885.4764 [M-H-Glc]^−^, 739.4232 [M-H-Glc-Rha]^−^, 593.3678 [M-H-Glc-2Rha]^−^, 431.1245 [M-H-2Glc-2Rha]^−^
36	Parispseudoside A	Cholestanol	1229.6029 [M + COOH]^−^	20.876	1037.5500 [M-H-Rha]^−^, 891.4866 [M-H-2Rha]^−^, 745.4190 [M-H-3Rha]^−^
37	(8R,9R,10S,6Z)-triol-octadec- 6-enoic acid/glyceryl *α*-mono-palmitate	Others	329.2348 [M-H]^−^	22.429	
38	7*β*-ol-sitosterol-3-O-Glc	Phytosterol	541.3452 [M-H]^−^	23.046	379.2784 [M-H-Glc]^−^
39	Polyphyllin VII	Isospirosteranol	1029.5424 [M-H]^−^	25.37	883.4873 [M-H-Rha]^−^, 737.4273 [M-H-2Rha]^−^, 591.9858 [M-H-3Rha]^−^, 429.1363 [M-H-3Rha-Glc]^−^
40	Cussonoside B/pregna-5,16-diene-3B-ol-20-one-3-O-Rha (1-2)-Rha (1-2)-[Rha (1-4)]-Glc	Pentacyclic triterpenoids	915.4648 [M-H]^−^	25.898	737.4158 [M-H-O-Glc]^−^, 591.3557 [M-H-O-Glc-Rha]^−^
41	Myrincitrin	Flavonoids	313.241 [M-H]^−^	28.267	277.2196 [M-H-CH_2_O]^−^
42	Polyphyllin II	Isospirosteranol	1013.5392 [M-H]^−^	29.057	867.4844 [M-H-Rha]^−^, 721.4232 [M-H-2Rha]^−^, 575.3578 [M-H-3Rha]^−^, 413.2257 [M-H-3Rha-Glc]^−^
43	Polyphyllin III	Isospirosteranol	883.4812 [M-H]^−^	29.411	737.4196 [M-H-Rha]^−^, 721.4225 [M-H-O-Rha]^−^, 575.3619 [M-H-2Rha]^−^, 413.1488 [M-H-2Rha-Glc]^−^
44	Polyphyllin I	Isospirosteranol	853.4645 [M-H]^−^	29.541	721.4166 [M-H-Ara]^−^, 575.3624 [M-H-Ara-Rha]^−^, 413.0833 [M-H-Ara-Rha-Glc]^−^
45	Polyphyllin V	Others	723.3857 [M-H]^−^	30.011	575.3649 [M-H-Rha]^−^, 397.1376 [M-H-Rha-Glc]^−^
46	7-O-Rha-kaempferol-3-O-Glc	Flavonoids	595.2918 [M-H]^−^	30.165	415.2354 [M-H-Glc]^−^
47	4,2′,4′-trihydroxy-chalcone	Flavonoids	255.2347 [M-H]^−^	35.700	237.2208 [M-H-OH]^−^

## Data Availability

The data used to support the findings of this study are included within the article.
